# GHS-R1a constitutive activity and its physiological relevance

**DOI:** 10.3389/fnins.2013.00087

**Published:** 2013-05-29

**Authors:** Yves Mear, Alain Enjalbert, Sylvie Thirion

**Affiliations:** ^1^CNRS, CRN2M UMR7286, Aix Marseille UniversityMarseille, France; ^2^Molecular Biology Laboratory, Conception Hospital, AP-HMMarseille, France

**Keywords:** ghrelin receptor, GPCR, constitutive activity, signaling pathway, PLC, β-arrestin

## Abstract

Abundant evidences have shown that ghrelin, by its binding to GHS-R1a, plays an important role for fundamental physiological functions. Increasing attention is given to the GHS-R1a unusually high constitutive activity and its contribution to downstream signaling and physiological processes. Here, we review recent lines of evidences showing that the interaction between ligand-binding pocket TM domains and the ECL2 could be partially responsible for this high constitutive activity. Interestingly, GHSR-1a constitutive activity activates in turn the downstream PLC, PKC, and CRE signaling pathways and this activation is reversed by the inverse agonist [D-Arg^1^, D-Phe^5^, D-Trp^7,9^, Leu^11^]-substance P (MSP). Noteworthy, GHSR-1a exhibits a C-terminal-dependent constitutive internalization. Non-sense GHS-R1a mutation (Ala204Glu), first discovered in Moroccan patients, supports the role of GHSR-1a constitutive activity in physiological impairments. Ala204Glu-point mutation, altering exclusively the GHSR-1a constitutive activity, was associated with familial short stature syndrome. Altogether, these findings suggest that GHS-R1a constitutive activity could contribute to GH secretion or body weight regulation. Consequently, future research on basic and clinical applications of GHS-R1a inverse agonists will be challenging and potentially rewarding.

## Introduction

The secretion of growth hormone (GH) by the anterior pituitary is under complex control. Small synthetic molecules termed GH secretagogues (GHS) are synthetic, peptidyl, and non-peptidyl molecules which possess strong and dose-dependent GH-releasing activity *in vivo* in several species and in humans (Cheng et al., 1989; Bowers et al., 1991; Cheng et al., 1991). The cloning of the GH secretagogue receptor (GHS-R1a, now called ghrelin receptor) in 1996 (Howard et al., 1996; Pong et al., 1996), led to the isolation of the endogenous ligand, ghrelin in 1999 (Kojima et al., 1999).

Ghrelin is a 28 amino acid peptide that differs from all other peptide hormones known by an octanoylation. This fatty acid modification is essential both for the binding to and activation of the receptor and for its pharmacokinetic properties (Kojima et al., 2001). Ghrelin strongly stimulates GH release in humans (Bowers et al., 1991), and it is much more potent than Growth Hormone-Releasing Hormone (GHRH) under similar conditions. Ghrelin potently increase food intake and weight gain, and also regulates energy homeostasis and metabolism following central and systemic administration (Castañeda et al., 2010; Stengel and Taché, 2012).

The GHS-R1a is a G protein-coupled receptor (GPCR) of 366 amino acids with the characteristic seven transmembrane-spanning domains (7TM receptor; for review Schwartz et al., 2006; Cruz and Smith, 2008). Signal transduction from the extracellular environment via 7TM receptors in general requires a conformational change from an inactive (R) to a active state (R^*^). Certain 7TM receptors are stabilized in an active conformation without any ligand present. The ability to propagate the intracellular signal in the absence of agonist is commonly known as constitutive activity.

Constitutive activity is described for mostly all GPCR, however for a large part of them the level of constitutive activity is very low (Arvanitakis et al., 1998; Smit et al., 2007).

The GHS-R1a exhibits unusual high constitutive activity (Holst et al., 2003) as it signal with ~50% of its maximal capacity in the absence of the agonist, ghrelin. This article aims to review the current knowledge on GHS-R1a ligand-independent constitutive activity and its functions.

## GHS-R1a and its constitutive activity

### Molecular basis

For several years, it has been noted that mostly all GPCRs exhibit intrinsic constitutive activity (Arvanitakis et al., 1998; Smit et al., 2007). In 1999, studies performed on the β_2_-adrenergic receptor suggested that this ligand-independent activity could involve an inward movement of the extracellular segments of the transmembrane domains (TMs) VI and VII toward TM III in the ligand-binding pocket (Elling et al., 1999).

Holst and colleagues performed a structural analysis of GHS-R1a and revealed the crucial role of an aromatic cluster formed by three residues (*Phe VI:16, Phe VII:06, and Phe VII:09)* on the inner face of the extracellular ends of GHS-R1a TMs VI and VII. Their close spatial proximity and the formation of this cluster allow GHS-R1a to stabilize in its active conformation in absence of agonist (Holst et al., 2004; Mokrosinski and Holst, 2010).

The residue in position *VI:16* is central for the constitutive activity level that can gradually be increased or decreased depending on the size and hydrophobic properties of the side chain of the amino acid. It has also been suggested that the aromatic residue *VI:16* may work as a tethered agonist located strategically at the interface between TM III TM VI and TM VII, blocking these extracellular TM segments in a conformation promoting GHS-R1a high constitutive activity (Schwartz et al., 2006).

A conserved aromatic lock crucial for GHS-R1a high basal signaling level is formed by the Trp *VI:13* and Phe *V:13* residues (Holst et al., 2004). The Trp *VI:13* (=Trp276) is located in the conserved motif CWxP in the middle of TM VI and is supposed to act as a global toggle switch model allowing the inward movement of this domain, and the GHS-R1a high basal activation level (Schwartz et al., 2006; Floquet et al., 2010).

Specific residues in the vinicity of this cluster have been proposed to orchestrate finely tuned microswitches critical for the activation level in absence of ligand (Holst et al., 2004; Valentin-Hansen et al., 2012).

In order to study the importance of this core peptide in the GHS-R1a constitutive activity, Gozé et al. introduced the mutation Trp276Ala and mutated the two surrounding amino acid residues Val131 and Ile134. Their results revealed that the mutation Trp276A1a dramatically impairs the ligand-independent activity whereas Val131Leu and Ile134Met highly increase GHS-R1a basal activity. According to these results, the three residues Trp276, Val131, and Ile134 could also significantly impact on GHS-R1a constitutive signaling (Gozé et al., 2010).

In 2006, Pantel et al. reported a mutation (Ala204Glu) in the extracellular loop II (ECL2) of the human GHS-R1a affecting selectively the ligand-independent activity (Pantel et al., 2006). The ECL2 structure function analysis revealed that by restricting this segment, and so possibly TM V/TM III, movements either by mutation or by ligand binding, reduces the constitutive signaling level (Mokrosinski et al., 2012), showing that the high GHS-R1a basal signaling level depends on the flexibility in these segments. Other studies showed that a single mutation or space generating a substitution in the GHS-R1a sequence or in the ligand peptide sequence can change the ligand properties from agonist to inverse agonist or from inverse agonist to agonist depending on the residue mutated (Holst et al., 2007; Els et al., 2012).

TM VI and TM VII movements into their inward-bend promoting the ligand-independent active conformation can be stoically blocked, using a modified substance P (MSP) inverse agonist, [D-Arg^1^, D-Phe^5^, D-Trp^7,9^, Leu^11^]-substance P (often referred in the literature as SPA, for substance P analog). The systematic analysis of this peptide structure-function relationship identified the C-terminal heptapeptide (fQwFwLL) as its active core, the D-Phe^5^ residue being apparently crucial for the inverse agonist property and the binding affinity (Holst et al., 2006). Unlike ghrelin that only interacts with the middle part of the ligand-binding pocket, the inverse agonist binds to an extended-binding pocket comprising all the seven TM domains of the receptor except for the first one. In addition, the space-generating mutants located relatively deep in the binding pocket at key positions within the TM III, TM IV, and TM V, upregulate the effects of MSP, suggesting that this molecule could prevent the spontaneous receptor activation across the binding pocket extend (Holst et al., 2006; Mokrosinski and Holst, 2010).

### Intracellular pathways and constitutive internalization

Inositol phosphate (IP) signaling pathway, through Phospholipase C (PLC) activation, was the first specifically associated with the GHS-R1a ligand-dependent activity (Adams et al., 1995; Lei et al., 1995; Chen et al., 1996; Petersenn, 2002) inducing intracellular calcium mobilization (Herrington and Hille, 1994). Consequently, this pathway has been investigated for determining the receptor constitutive signaling. PLC activation was demonstrated comparing heterologous HEK-293 and COS-7 cells overexpressing the GHS-R1a to cells transfected with the motilin receptor (that is the closest GPCR homolog of GHS-R1a without constitutive activity; Holst et al., 2003). The unusualy high GHS-R1a ligand-independent signaling level was similar to that of the most famous highly constitutively active GPCR, the virally encoded ORF74 receptor. To note, this paper allowed characterizing MSP as a full GHS-R1a inverse agonist. Indeed, this compound inhibits the GHS-R1a constitutive ligand-independent IP accumulation and decreases the IP level to that of cells transfected with the empty vector (Holst et al., 2003). Lau et al. reported that GHS-R1a constitutive activity could reduce apoptosis in HEK-293 overexpressing the (seabream) sbGHS-R1a, through PKC-dependent caspase-3 inhibition (Lau et al., 2009).

**Figure 1:**
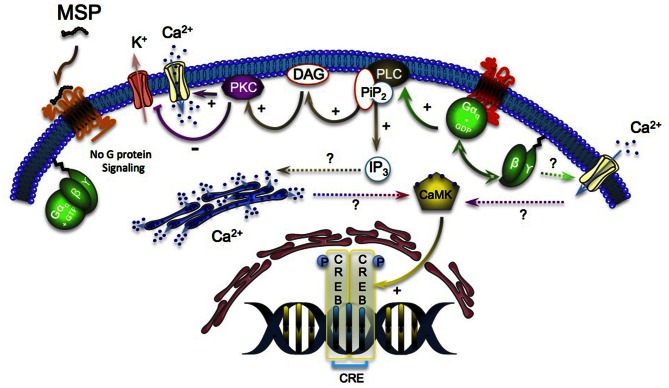
**GHS-R1a constitutive activity leads to the dissociation of α and βγ subunits of heterotrimeric G-proteins.** The free Gq subunit activates the PLC that cleaves PiP_2_ into IP3 and DAG. DAG activates the PKC, which in turn actives the Ca^2+^ channels and inhibits the K^+^ channels (continuous line). The effects of IP3 on the intracellular Ca^2+^ mobilization and the origin of the Ca^2+^ that activates the Ca^2+^ calmodulin kinase (CaMK) remain unclear and will need further investigations (dotted line). The binding of the inverse agonist MSP to the GHS-R1a inhibits the G-protein signaling and decreases the IP3 (via PLC) and CRE pathway (via phosphorylated CREB) constitutive activation.

Gq/11-coupled GHS-R1a constitutive activity also resulted in a dose-dependent but ligand-independent increase in the CRE luciferase reporter while the full inverse agonist MSP partially reversed this effect. To note, heterotrimeric Gq/11 protein-coupled receptors have previously been reported to phosphorylate the cAMP responsive element-binding protein (CREB) by activating the CRE pathway as a result of calcium/calmoduline kinase IV (CaMK IV) and/or protein kinase C (PKC) activation (Matthews et al., 1994; Poulin et al., 2009) (Figure 1). Finally, performing serum responsive element (SRE) reporter assay on HEK-293 cells transfected with GHS-R1a revealed a 10-fold increase in the ligand-independent signaling compared to the cells transfected with the empty plasmid.

While many other signaling pathways have been shown to play a role in the GHS-R1a ligand-dependent activation, the GHS-R1a ligand-independent activation has not been investigated. Indeed, Gi/o heterotrimeric pathway was clearly associated with beta-arrestin-mediated ERK1/2 activation following GHS-R1a activation by ghrelin (Camiña et al., 2007). Similarly, c-Src that is involved in the GHS-R1a ghrelin-dependent Akt activation (via Gi/o-protein; Lodeiro et al., 2009) has not been studied for the high basal level associated signaling pathways.

Since GHS-R1a exhibits an unusually high constitutive activity, it could be hypothesized that the downstream signaling level could reflect the membrane expression level. In this context, a better understanding of the mechanisms underlying and modulating its plasma membrane expression was necessary. To this end, GHS-R1a was tagged using M2 anti-FLAG antibody labeling, thus allowing following the intracellular movement of GHS-R1a. These experiments revealed a GHS-R1a constitutive ligand-independent internalization and the receptor could be trapped at the cell surface by the inverse agonist MSP. The punctiform receptor intracellular labeling co-localizes with clathrin-coated vesicles and recycling endosome markers (Holst et al., 2003).

Unlike the GHS-R1a, GPR-39 receptor, a member of the ghrelin receptor family, is not constitutively internalized but it displays a high ligand-independent signaling level (Holst et al., 2003). Based on these observations, Holliday et al. developed an elegant approach by switching the GHS-R1a C tail with that of the GPR-39. The chimera, named GhR-39, was constitutively active through the PLC pathway but its internalization was impaired. Components supporting the constitutive activity could be necessary but not sufficient for GHS-R1a endocytosis and additional regulatory elements in the C-terminal domain may be involved (Holliday et al., 2007).

GHS-R1a constitutive internalization requires the sequential activation of the monomeric G proteins Rab5 and Rab11. Rab proteins control various important cellular processes, such as endocytosis, trafficking, endosome fusion. and exocytosis (Seachrist and Ferguson, 2003). These proteins regulate vesicle transport and fusion with specific target compartments, the early endosomes for Rab5, and the endosomal recycling compartments such as the perinuclear recycling compartment (PNRC) for Rab11. MSP-induced GHS-R1a membrane plasma anchorage is blocked by the constitutive expression of GTP-binding mutants of Rab_s_ (Rab5 Gln79Leu or Rab11 Gln70Leu) confirming that MSP naturally inhibits GHS-R1a internalization rather than activates GHS-R1a neosynthesis and trafficking (Holliday et al., 2007).

**Figure 2:**
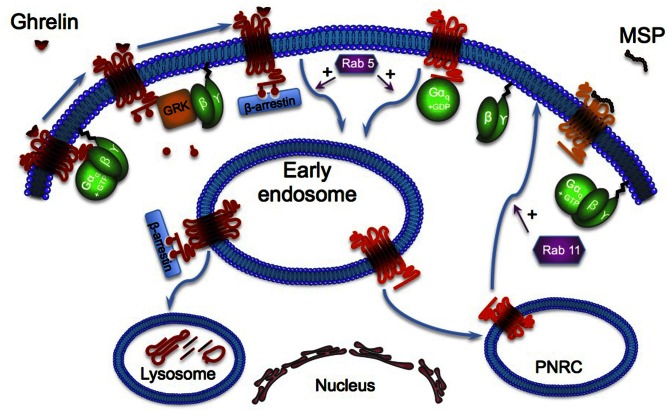
**GHS-R1a activation by its endogenous ligand ghrelin leads to the dissociation of α and βγ subunits of heterotrimeric G-proteins.** The βγ dimer recruits G-protein-receptor kinases (such as GRK_2_ or GRK_3_) to the receptor, where they phosphorylate the agonist-bound receptor. These phosphorylations lead to the recruitment of β-arrestins and activation of the monomeric G-protein RAb_5_ that targets the receptor to early endosomes. Then the receptor is addressed to the lysosome for degradation. GHS-R1a constitutive activation recruits Rab_5_ without activation of the GRK, inducing subsequent phosphorylation and β-arrestin recruitment. This constitutive activation of Rab_5_ explains the receptor ligand-independent internalization that is first addressed to the early endosome then to the PNRC (perinuclear recycling compartment) where it activates the monomeric G-protein Rab_11_.

β-arrestin recruitment has been obviously investigated as it appears as the most widely standard adaptor for GPCR endocytosis (Lefkowitz, 1998) (Figure 2). Only ghrelin stimulation induces GHSR-1a phosphorylation and β-arrestin 2 recruitment. Besides, dominant-negative β-arrestin 2 construct, which competes for clathrin interaction, does not inhibit the constitutive endocytosis, supporting the hypothesis of a GHS-R1a β-arrestin-independent constitutive internalization. These results suggest that the aromatic residue *VI:16*, already mentioned above, could act as “tethered biased agonist” rather than “tethered agonist” because it blocks the receptor in a conformation that only induces the Gq/11 protein activation without β-arrestin 2 recruitment (Shukla et al., 2011; Reiter et al., 2012). To note, β-arrestin 2 recruitment has been reported to active the MAPK pathway. In this context, the absence of β-arrestin 2 recruitment in basal condition has been proposed to explain the absence of MAPK pathway activation (Holliday et al., 2007).

Purified GHS-R1a monomers in a lipid disc showed that the ghrelin receptor *per se* activates Gq/11 in the absence of agonist, and that GHS-R1a constitutive activity is an intrinsic property of the protein and is not influenced by its cellular environment (Damian et al., 2012). In this context, the receptor isolated in lipid discs recruits arrestin-2 in an agonist-dependent manner (Mary et al., 2012), whereas it interacts with μ-AP2 (plasma membrane-localized clathrin adaptor subunit) in the absence of ligand or in the presence of ghrelin (Damian et al., 2012). Thus, μ-AP2 could be involved in the basal regulation of GHS-R1a trafficking.

## Physiological relevance

Many types of GPCR display a high ligand-independent signaling *in vitro* (Seifert and Wenzel-Seifert, 2002). Among them, GHS-R1a has been shown to display both the highest basal activation of Gq/11 (about 50% of its maximal activity) *in vitro* (Holst et al., 2004), and substantial basal signaling for food intake and weight control *in vivo* (Petersen et al., 2009; Els et al., 2012). Pantel et al., who reported naturally occurring mutations in the GHS-R1a sequence, have first shown a putative link with physiological impairments in 2006. They reported a substitution mutation located within the first GHS-R1a exon, predicting the substitution of Alanine by Glutamate (Ala204Glu) in two independent Moroccan families. This missense mutation is located in the GHS-R1a ECL2 and affects a fully conserved amino acid. HEK-293 cells stably transfected with WT and Ala204Glu-mutant GHS-R1a showed, using POU1F1-luciferase reporter assay that this mutation selectively abolishes the GHS-R1a ligand-independent signaling without altering the Ghrelin-dependent activity. Noteworthy, mutations altering exclusively the constitutive activity are associated with familial short stature syndrome and the latter can be partially reversed with GH treatment (Pantel et al., 2006, 2009; Inoue et al., 2011). Wang reported another uncharacterized GHS-R1a mutation affecting the Phe 279 residue (Phe *VI:16*) recently identified as being of particular interest for GHS-R1a constitutive activity. Noteworthy, the phenotype of patients expressing this mutation is characterized by an increased obesity and short stature (Wang, 2004). Regarding these different but functionally similar mutations, Holst and Schwartz proposed that the absence of GHS-R1a constitutively active signaling results in a syndrome, characterized, not only by a short stature, but also by obesity (Holst and Schwartz, 2006). This suggestion has been confirmed recently using icv MSP administration that significantly decreased the food intake, body weight, and neuropeptide Y (NPY) and uncoupling protein 2 (UCP2) gene expression in the hypothalamus (Petersen et al., 2009).

Ligand-dependent GHS-R1a heterodimerizations have been reported for the subtype 1 dopamine receptor that increases dopamine signaling (Jiang et al., 2006), the somatostatin receptor-5 that regulates insulin release (Park et al., 2012) and the melanocortin-3 receptor that is involved in body weight regulation and energy balance (Rediger et al., 2011, 2012). Also, the dimerization of the GHS-R1a with the dopamine D2 receptor has recently been shown to regulate appetite (Kern et al., 2012).

Its dimerization with the melanocortin-3 receptor, the D1 receptor, and the newly discovered 5-HT_2C_ receptor may be central in modulating and controlling GHS-R1a-mediated downstream signaling and subsequent satiety and appetite signaling, as well as the rewarding and motivational aspects of food intake (Schellekens et al., 2013). The understanding of the underlying mechanisms leading to these activations may ultimately lead to the development of new therapeutic strategies.

On the other hand, ghrelin and GHS-R1a knockout mice explorations may allow the emergence of new regulatory properties of the ghrelin receptor constitutive activity. For example, GHS-R1a constitutive activity increases limbic seizures in rodents, the endogenous ligand being naturally anticonvulsive (Portelli et al., 2012). In the mouse brain, deficits in spontaneous receptor activity cause marked functional impairment in learning and memory (Albarran-Zeckler et al., 2012). GHS-R1a signaling is necessary for hippocampal-dependent learning and habituated feeding responses (Davis et al., 2011). Ghrelin plays also a role in sleep whereas the GHS-R1a will be more implicated in arousal (Esposito et al., 2012).

To move beyond putative redundant compensation mechanisms, further *in vivo* investigations targetting the receptor constitutive activity with appropriate pharmacological tools (Mokrosinski and Holst, 2010; Sivertsen et al., 2011) would be needed to determine the underlying physiological functions linked to this constitutive activity.

## Conclusion

Abundant evidence currently indicates that ghrelin, by its binding to GHS-R1a, plays a role in various physiological functions that led to the development of clinical trials to translate basic research findings to human disease treatment and diagnosis (for review Akamizu and Kangawa, 2012). Concerning the GHS-R1a constitutive activity, most of the studies performed so far focused on the molecular aspects. Even if some physiological relevance are emerging for food intake regulation (see paragraph above), further studies remain necessary to confirm the role played by the GHS-R1a constitutive activity in the regulation of the GH axis. Pituitary somatotroph adenomas express higher GHS-R1a transcript and protein levels than normal pituitary (Korbonits et al., 1998; Barlier et al., 1999). Moreover, Pantel et al. (2006) have shown that a reduced GHS-R1a constitutive activity impairs the GH secretion. Thus, it should be interesting to test if the GHS-R1a constitutive activity disrupts the GH hypersecretion observed in acromegalic patients with somatotroph tumors (Acunzo et al., 2008; Roche et al., 2012).

The signaling pathways associated with this high constitutive activity should also be addressed. Knowing that the heterogeneity of the coupling on the signaling varies depending on the tissue or cell type, it is crucial to address now these questions in physiological or pathophysiological models. Depending on the model, does the GHS-R1a ligand-independent CRE activation pass through the calcium/calmodulin kinase? And/or via the cAMP/PKA pathways?

These issues are part of the many questions pending in this field. Some of them may support the development of clinical applications of GHS-R1a inverse agonists in physiological disorders in the future, allowing developing novel and unique therapies for various disorders, including intractable and serious diseases. Indeed, research on basic and clinical applications of GHS-R1a inverse agonists will be challenging and potentially rewarding.

### Conflict of interest statement

The authors declare that the research was conducted in the absence of any commercial or financial relationships that could be construed as a potential conflict of interest.
